# Dynamic Alterations in Salivary Microbiota Related to Dental Caries and Age in Preschool Children With Deciduous Dentition: A 2-Year Follow-Up Study

**DOI:** 10.3389/fphys.2018.00342

**Published:** 2018-04-04

**Authors:** Lei Xu, Xi Chen, Yuan Wang, Wen Jiang, Sa Wang, Zongxin Ling, Hui Chen

**Affiliations:** ^1^Department of Conservative Dentistry and Periodontics, The Affiliated Hospital of Stomatology, College of Medicine, Zhejiang University, Hangzhou, China; ^2^State Key Laboratory for Diagnosis and Treatment of Infectious Diseases, Collaborative Innovation Center for Diagnosis and Treatment of Infectious Diseases, The First Affiliated Hospital, College of Medicine, Zhejiang University, Hangzhou, China

**Keywords:** dynamic alteration, salivary microbiota, caries, age, deciduous dentition, follow-up study, Illumina MiSeq, 16S rDNA

## Abstract

Dynamic alterations in oral microbiota are closely related to the development of dental caries;however, changes in salivary microbiota during this process have not been extensively studied. In addition, increasing evidence suggests that oral microbiome profiles differ according to dentition stages, but it is unclear whether they change with age during the same dentition, such as deciduous dentition. These two aspects were investigated in a 2-year follow-up study, and caries-free preschool children with complete deciduous dentition were enrolled. Saliva was collected and oral examination was conducted at the beginning of this trial, and then every subsequent 6 months for a total of five time points (T0, T1, T2, T3, and T4). Based on the clinical examination of teeth at the end of the trial, subjects were divided into health-to-health (H-H, *N* = 11) and health-to-caries (H-C, *N* = 12) groups at every time point. A total of 115 saliva samples from 23 subjects was detected by sequencing 16S rDNA V3-V4 hypervariable regions with the Illumina MiSeq platform to obtain microbiome profiles, and 100 samples finally passed quality control for further analyses. A total of 4,328,852 high-quality sequencing reads passed quality-control testing, representing 14 phyla, 27 classes, 43 orders, 67 families, and 127 genera. An α diversity analysis showed that salivary microbial diversity was similar in all groups, and a β diversity analysis showed that salivary microbial community structure changed with dental caries. Linear discriminant analysis effect size (LEfSe) analysis revealed that the abundance of the genera *Atopobium, Megasphaera*, and *Veillonella* increased significantly, while that of the genera *Shuttleworthia* and *Rothia* decreased significantly with the development of dental caries. *Megasphaera* and *Veillonella* were enriched at the early stage of deciduous dentition whereas *Peptococcus, Rothia*, and *Treponema* were enriched at the later stage. The core microbiome in the H-H and H-C groups comprised 26 and 29 genera, respectively, with statistical differences observed in 11 shared core genera. These results provide new insights into variations in the salivary microbiome related to dental caries and age in the deciduous dentition period.

## Introduction

Microbes reside in different parts of the human body, and the number of them to human cells is found to be much closer to 1:1, rather than at least 10:1 as commonly reported (Sender et al., 2016). Indeed, humans are regarded as superorganisms that possess a combination of microbial and human attributes (Gill et al., 2006). The oral cavity is one of the most important habitats for microorganisms and considerable efforts have been made to characterize human oral microbiotas (He and Shi, 2009). The mouth is a constantly changing habitat for microbes, in part due to the emergence and later replacement of deciduous teeth, as well as diet, environment, and host genetics, among other factors (Human Microbiome Project, 2012). Studies have shown that oral microbial community composition changes throughout life, from neonates to primary dentition, mixed dentition, and permanent dentition of young adults, adults, and the elderly (Crielaard et al., 2011; Lif Holgerson et al., 2015; Xu et al., 2015; Shi et al., 2016). However, age-related changes in the oral microbiome for a given dentition status, such as deciduous dentition, have not been adequately addressed.

Oral microbes contribute to the maintenance of oral homeostasis but also play an important role in systemic and oral diseases, including dental caries and periodontal disease (Chen and Jiang, 2014). The development of dental caries involves interactions between microbial pathogens and the host's diet and immune mechanisms. Given the limitations of the specific plaque hypothesis (Loesche, 1979), early studies on dental caries have mainly focused on identifying primary pathogens including *Lactobacilli* and *Streptococcus mutans* (Van Houte, 1993). The advent of high-throughput sequencing of 16S rDNA has enabled examination of the microbiome. The microbiome is considered to be an ecological community of commensal, symbiotic, and pathogenic microorganisms that share our body space. In addition, the oral microbiota naturally colonizes the oral cavity and can counterbalance acid production from the dietary intake of carbohydrates, for example, by ammonia production from arginine or urea (Burne and Marquis, 2000). Dysbiosis of the oral microbiota occurs when excessive and frequent acid production exceeds its buffering capacity, which increases the risk of demineralization of the tooth surface and can lead to dental caries (Tanner et al., 2018).

Dental caries is prevalent in Chinese children (Wang et al., 2002; Hu et al., 2011), and many researchers have focused on it and identified the oral microbes in children with high-throughput sequencing to explore the relationship between dental caries and the oral microbiota composition. The presence of *Streptococcus, Veillonella, Actinomyces, Granulicatella, Leptotrichia*, and *Thiomonas* in dental plaques was found to be significantly associated with dental caries in children between the ages of 3 and 6 years (Ling et al., 2010); another study reported that *Streptococcus, Granulicatella*, and *Actinomyces* counts were elevated in dental plaques of children with severe caries (Jiang et al., 2013). These authors also demonstrated that eight genera (*Cryptobacterium, Lactobacillus, Megasphaera, Olsenella, Scardovia, Shuttleworthia, Cryptobacterium*, and *Streptococcus*) were abundant in cavitated dentin lesions in children between 3 and 7 years old (Jiang et al., 2014). An additional study of children younger than 30 months showed that *Streptococcus* and *Veillonella* were associated with the development of caries whereas *Leptotrichia, Selenomonas, Fusobacterium, Capnocytophaga*, and *Porphyromonas* were linked to its absence (Xu et al., 2014). However, variations related to caries in the salivary microbiome have been inadequately studied and more work is required.

In addition, most studies to date have been cross-sectional; as such, it is unclear whether there were initial differences in microbial communities between subjects with caries and controls when both groups were caries-free, which probably could explain the discrepancies in the findings. To address this issue, we carried out a longitudinal follow-up, case-control study to eliminate the potential biases caused by age, gender, and sample selection.

The salivary microbiota in preschool children with complete deciduous dentition was analyzed by high-throughput sequencing of the 16S rDNA V3–V4 hypervariable regions with the Illumina MiSeq platform. We studied variations in the salivary microbiota related to caries by comparing H-H and H-C groups, while looking at changes in the salivary microbiota with age during the deciduous dentition period by comparing H-H groups between T0 and T4 time points. Our results provide novel insights into the possible causes of dental caries and age-related microbial alterations in deciduous dentition.

## Materials and methods

### Ethics statement

This study was carried out according to the recommendations of the ethics committee of the Affiliated Hospital of Stomatology, Medical College, Zhejiang University. Written informed consent was obtained from parents or guardians of all participants prior to recruitment in accordance with the Declaration of Helsinki. Detailed written informed consent was provided in Presentation 2, in the Supplementary Material.

### Subject selection and clinical screening

The study subjects were preschool children with complete healthy deciduous dentition and without dental restoration who were recruited from the same kindergartens in Lin'an County, Hangzhou City, Zhejiang Province, China. The exclusion criteria were as follows: the use of antibiotics, probiotics, synbiotics, or additional fluoride to fluoridated toothpaste within the prior 3 months; apparent active bacterial or viral infection in any part of the body (Ling et al., 2010); visually detectable enamel or dentin hypoplasia; and eruption of permanent teeth during the study. Subjects were instructed to brush their teeth with fluoridated toothpaste. Dental caries was diagnosed based on the criteria of the International Caries Detection and Assessment System (ICDAS) (Shivakumar et al., 2009). The caries status of each individual was determined by the same two experienced dentists who performed a whole oral clinical examination. According to ICDAS criteria, a code greater than 2 was considered to reflect the presence of dental caries in this study.

Sixty subjects were initially recruited and 23 remained at the end of the study. Subjects were examined at five time points: T0 (baseline, at the beginning of the study), T1 (6 months later), T2 (12 months later), T3 (18 months later), and T4 (24 months later, at the end of the study). The average age of the subjects was 47.5 months (SD = 2.2) at the baseline. The oral clinical examinations were implemented at each time point and revealed an increasing number of subjects with dental caries over time, with 12 subjects eventually developing caries. The 23 remaining individuals were divided into two groups: the health-to-health (H-H) group (*N* = 11), which included individuals with no caries and no dental restoration at the end of the study; and the health-to-caries (H-C) group (*N* = 12), which included individuals with caries and/or dental restoration at the end of the study. Saliva samples were collected at all five time points such that there were 10 groups in total (H-H-T0, H-C-T0, H-H-T1, H-C-T1, H-H-T2, H-C-T2, H-H-T3, H-C-T3, H-H-T4, and H-C-T4).

### Sample collection and preparation

Sampling was performed in the morning before brushing, gargling, and breakfast. Subjects were asked to open their mouth and stop swallowing to allow saliva to flow into a sterile cryogenic vial (Corning Inc., Corning, NY, USA). A 2-ml sample of spontaneous, non-stimulated whole saliva was obtained from each individual, which was immediately frozen in liquid nitrogen and stored at −80°C until use (Ling et al., 2010).

### Total bacterial genomic DNA extraction

Microbes were pelleted from saliva samples by centrifugation at 13,000 × g for 10 min. The pellets were resuspended in lysis buffer, homogenized with 200 mg of glass beads (0.1 mm) for 2 min using a FastPrep Mini-Beadbeater (Thermo Fisher Scientific, Waltham, MA, USA), and incubated at 56°C for 1 h. DNA was extracted with the QIAamp DNA Mini kit (Qiagen, Hilden, Germany) according to the manufacturer's instructions. The DNA concentration was measured with a NanoDrop ND-1000 spectrophotometer (Thermo Fisher Scientific), and the sample was stored at −20°C until use.

### PCR and sequencing

The bacterial 16S rDNA V3-V4 hypervariable regions were amplified by PCR using universal bacterial primers 338F (5′-ACTCCTACGGGAGGCAGCAG-3′) and 806R (5′-GGACTACHVGGG- TWTCTAAT-3′). The PCR mixture (final volume: 20 μl) contained 4 μl of 5× reaction buffer (TransStart FastPfu Buffer; TransGen Biotech, Beijing, China), 10 ng bacterial genomic DNA, 0.8 μl each primer, 0.4 μl DNA polymerase (TransStart FastPfu DNA Polymerase; TransGen Biotech), and 2 μl of 2.5 mM dNTPs. The reaction was carried out on an ABI GeneAmp 9700 instrument (Applied Biosystems, Foster City, CA,USA) under the following conditions: 95°C for 3 min; 27 cycles of 95°C for 30 s, 55°C for 30 s, and 72°C for 45 s; and 72°C for 10 min. The PCR products were separated by 2% agarose gel electrophoresis, and fragments with a size of ~450 base pairs (bp) were purified with the AxyPrep DNA Gel Extraction Kit (Axygen Biosciences, Union City, CA, USA) and quantified with a QuantiFluor-ST hand-held fluorometer (Promega, Madison, WI, USA). PCR products were sequenced on a MiSeq system (Illumina, San Diego, CA, USA).

### Data processing

Raw fastq files were demultiplexed and quality filtered using QIIME v.1.17 (Caporaso et al., 2010) according to the following criteria: (i) reads were truncated at any site with an average quality score <20 over a 50-bp sliding window while discarding truncated reads shorter than 50 bp; (ii) exact barcode matching, two-nucleotide mismatch in primer matching, and reads containing ambiguous characters were removed; and (iii) only sequences overlapping by more than 10 bp were assembled according to their overlap sequence. Reads that could not be assembled were discarded. Operational taxonomic units (OTUs) with a 97% similarity cutoff were clustered using UPARSE v.7.1 (http://drive5.com/uparse/) and chimeric sequences were identified and removed using UCHIME. The taxonomy of each 16S rRNA gene sequence was analyzed with RDP Classifier (http://rdp.cme.msu.edu/) against the Silva (SSU115) 16S rRNA database using a confidence threshold of 70% (Amato et al., 2013).

### Sequence analysis

Demographic data were compared with the χ^2^ test (gender ratio) and Student's *t*-test (age). Differences in αdiversity indices and microbial relative abundance between H-H and H-C groups at five time points were calculated with the non-parametric Mann-Whitney *U*-test. The Wilcoxon signed-rank test was used to examine differences in αdiversity indices between H-H-T0 and H-H-T4 groups. All analyses were performed using SPSS v.19.0 software (SPSS Inc., Chicago, IL, USA), and *P* < 0.05 was considered statistically significant. Principle coordinate analysis (PCoA) was performed based on the weighted UniFrac distance to visualize similarities in the microbial structure between groups. Differences in features between the groups were evaluated based on linear discriminant analysis effect size (LEfSe) (Segata et al., 2011); the threshold logarithmic linear discriminant analysis score for discriminative features was 2.0.

### Data sets are in a publicly accessible repository

The raw reads generated in this study were deposited into the NCBI Sequence Read Archive (SRA) database under Accession Number SRP125517.

## Results

### Study population

Of the 60 individuals initially recruited for this study, 23 were available at each time point. Participants were lost to follow-up due to failure to attend their appointment, or because they had moved to another location, used antibiotics, or experienced eruption of permanent teeth, among other reasons. A total of 115 saliva samples were obtained from the 23 subjects. The demographic characteristics of the study population are shown in Table 1. At the beginning of study, the average age of subjects was 47.5 months old (SD = 2.2), and there was no statistical difference between the H-H and H-C groups in terms of age or sex ratio. Because the number of sequencing reads was low for some samples, we ultimately analyzed 100 samples. The number of microbiological samples obtained per visit was as follows: T0, *n* = 23; T1, *n* = 17; T2, *n* = 18; T3, *n* = 19; and T4, *n* = 23. There were three, five, nine, and 12 subjects with dental caries at T1, T2, T3, and T4, respectively (Table S1), and dfs index is also provided in Table S1.

**Table 1:** Demographic characters of the involved participants at baseline.

**Groups**	**Characters**	
	**Age (months, $\bar{\text{x}}$ ± S)**	**Gender (Male/Female, *n* & %)**
H_H group	47.4 ± 2.9	7/4 (63.6/36.4)
H_C group	47.6 ± 1.2^a^	5/7 (41.7/58.3)^b^
Total subjects	47.5 ± 2.2	12/11 (52.2/47.8)

a Student's t-test P = 0.210;

b *Chi-Square test P = 0.292*.

### Sequencing output and microbiota composition

Good's coverage was >0.998 for each sample in all 10 groups, indicating that the identified 16S rDNA sequences represented the vast majority of bacterial sequences present in the saliva samples. The richness of the saliva bacterial community was estimated with rarefaction curves (Figure S1), the shape of which indicated that bacterial richness was reflected by these sequences. The combination of these two indicators suggested that the sequencing depth was sufficient and that additional sampling was not required.

High-throughput Illumina MiSeq sequencing of saliva samples yielded 5,035,663 reads; 4,328,852 (86.0%) high-quality reads passed quality control for further analyses. An average of 43,289 reads was obtained for each of the 100 samples (SD = 4.911; range: 34,793–55,020), all of which were >400 bp with an average length of 445 bp per read (SD = 1.4; range: 439–448 bp). At a 97% similarity level, the reads were clustered into 401 OTUs representing 14 phyla, 27 classes, 43 orders, 67 families, and 127 genera.

**Figure 1:**
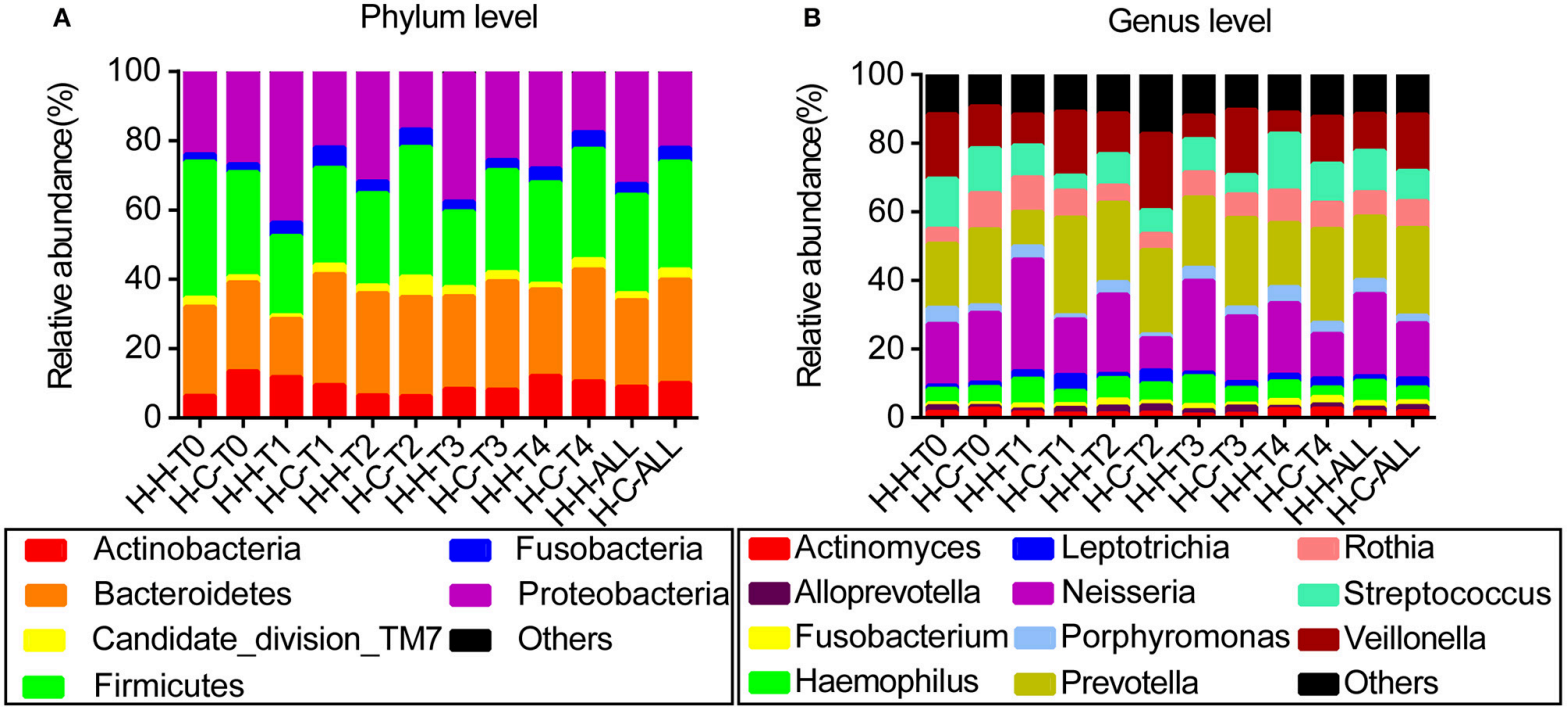
Distribution of dominant microbes at different taxonomic levels. The dominant taxa (>1% relative abundance) in each level are shown. **(A)** Distribution at the phylum level. **(B)** Distribution at the genus level.

When averaged over all time points, the reads were dominated by six phyla, namely Firmicutes (29.7%), Bacteroidetes (27.3%), Proteobacteria (27.1%), Actinobacteria (9.4%), Fusobacteria (3.6%), and Candidate division TM7 (2.5%); these constituted 99.6% of the whole microbiota, with other phyla detected in relatively low proportions (Figure 1A). The reads were dominated by 11 genera—namely *Prevotella* (21.9%), *Neisseria* (20.0%), *Veillonella* (13.5%), *Streptococcus* (10.5%), *Rothia* (7.5%), *Haemophilus* (5.2%), *Porphyromonas* (3.2%), *Leptotrichia* (2.0%), *Actinomyces* (1.7%), *Fusobacterium* (1.5%), and *Alloprevotella* (1.4%)—that constituted 88.4% of the whole microbiota (Figure 1B). Information regarding the distribution of dominant microbes in different groups at the phylum and genus levels (relative abundance, mean ± SD) is provided in Tables S2, S3, respectively.

### Comparison of salivary microbial diversity and community structures between the H-H and H-C groups

The Ace, Chao, Shannon, and Simpson alpha diversity indices are displayed in Table 2, and they did not differ between H-H and H-C groups at T0 and T4 (Figure S2), indicating that dental caries had no effect on the richness and evenness of the salivary microbiome. This was substantiated by the fact that there were also no differences between the two groups at the other three time points (Figure S2). The high similarity of the salivary microbiomes of the H-H and H-C groups was also revealed by Venn diagrams (Figure S3), which showed a high degree of overlapping OTUs; 314 OTUs were common between the H-H-T0 and H-C-T0 groups and accounted for 92.4 and 93.2% of their total OTUs, respectively. In addition, 324 OTUs were shared between the H-H-T4 and H-C-T4 groups, accounting for 92.6 and 93.9% of their total OTUs, respectively. At the remaining three visits, ~90% of the total OTUs were shared between the two groups.

**Table 2:** Alpha diversity indices for salivary microbiome in each group.

**Group**	**Ace**	**Chao**	**Shannon**	**Simpson**
	**Mean** ± **SD**	**Mean** ± **SD**	**Mean** ± **SD**	**Mean** ± **SD**
H-C-T0	246.5 ± 29.1	245.5 ± 31.5	3.16 ± 0.36	0.090 ± 0.036
H-C-T0	250.3 ± 20.0	251.6 ± 20.8	3.17 ± 0.18	0.089 ± 0.020
H-H-T1	254.8 ± 12.2	255.9 ± 16.2	3.09 ± 0.28	0.109 ± 0.050
H-C-T1	255.3 ± 17.1	256.1 ± 17.8	3.37 ± 0.21	0.072 ± 0.166
H-H-T2	252.8 ± 24.3	256.5 ± 19.3	3.18 ± 0.41	0.102 ± 0.081
H-C-T2	236.1 ± 23.7	235.2 ± 23.2	3.17 ± 0.44	0.093 ± 0.044
H-H-T3	250.6 ± 22.0	254.2 ± 24.3	3.07 ± 0.30	0.111 ± 0.051
H-C-T3	259.3 ± 22.6	260.2 ± 20.4	3.24 ± 0.27	0.091 ± 0.034
H-H-T4	261.9 ± 22.7	265.3 ± 23.0	3.18 ± 0.21	0.099 ± 0.037
H-C-T4	251.7 ± 18.1	252.4 ± 17.6	3.34 ± 0.22	0.075 ± 0.019

**Figure 2:**
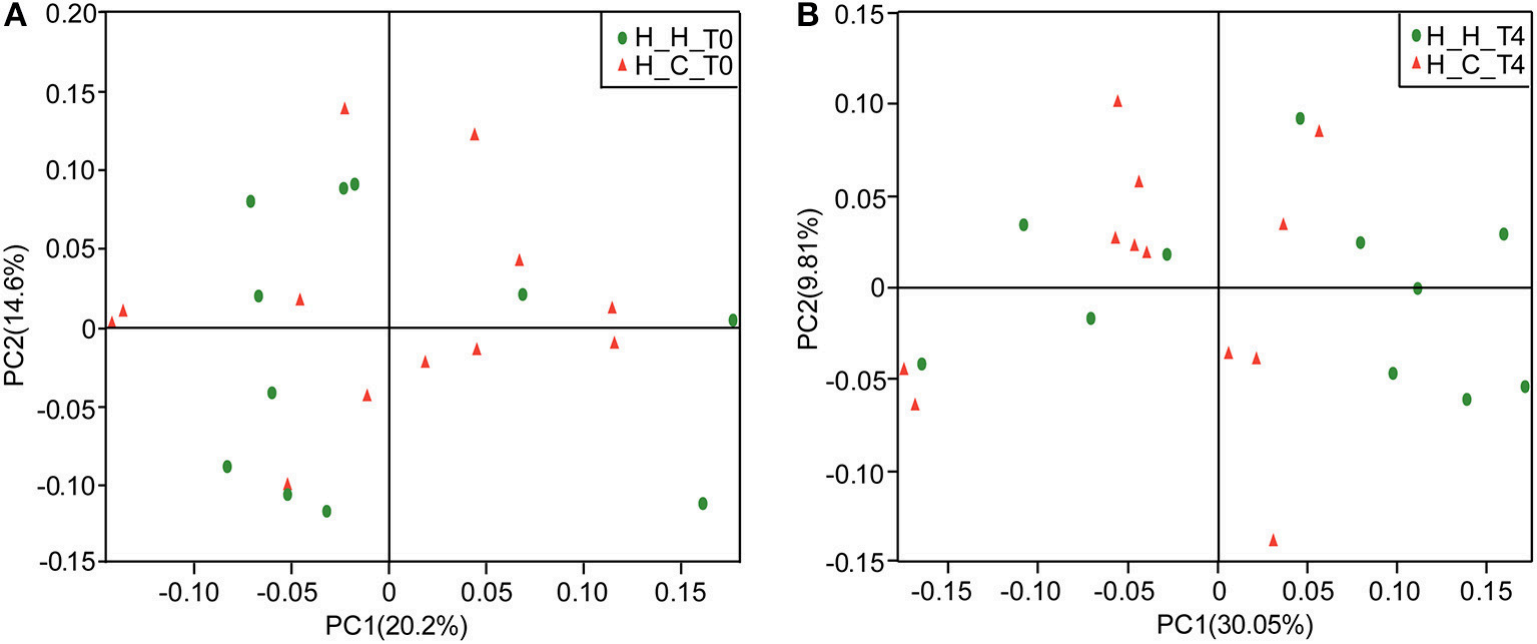
PCoA based on weighted UniFrac distances at the OTU level at 97% identity. Each sample is represented by a dot. Green balls represent the H-H samples. Red triangles represent the H-C samples. **(A)** The samples did not form well-separated clusters at T0, suggesting that the microbial community structures are similar between groups when they are all caries-free. **(B)** Separation was observed at T4, suggesting that the microbial community structures were different between groups after subjects in the H-C group suffered caries.

The overall bacterial community structures of the two groups were compared. PCoA plots based on weighted UniFrac distance measurements (β diversity) did not form well-separated clusters at T0 (Figure 2A), which was further confirmed by analysis of similarities (ANOSIM, *R* = 0.0081, *P* = 0.338), suggesting that the microbial community structures were similar between groups when they were all caries-free. However,separation was observed at T4 (Figure 2B). Furthermore, ANOSIM test revealed a marked difference between the H-H and H-C groups at T4 (*R* = 0.1638, *P* = 0.018), indicating that the microbial structures in healthy and caries groups were different.

### Comparison of the salivary microbial diversity and community structure between the H-H-T0 and H-H-T4 groups

At the beginning of study, the average age of subjects in the H-H-T0 group was 47.4 months old (SD = 2.9) and the subjects were in the early stage of deciduous dentition. Saliva samples were obtained from the same subjects 2 years later (H-H-T4 group) when they were in a later stage of deciduous dentition. No statistical differences were observed in the Ace, Chao, Shannon, and Simpson alpha diversity indices between the H-H-T0 and H-H-T4 groups (Figure S4), and the Venn diagram analysis showed a high degree of overlap in their OTUs (Figure S5). A total of 325 OTUs were shared by the H-H-T0 and H-H-T4 groups, accounting for 95.6 and 92.9% of their total OTUs, respectively.

**Figure 3:**
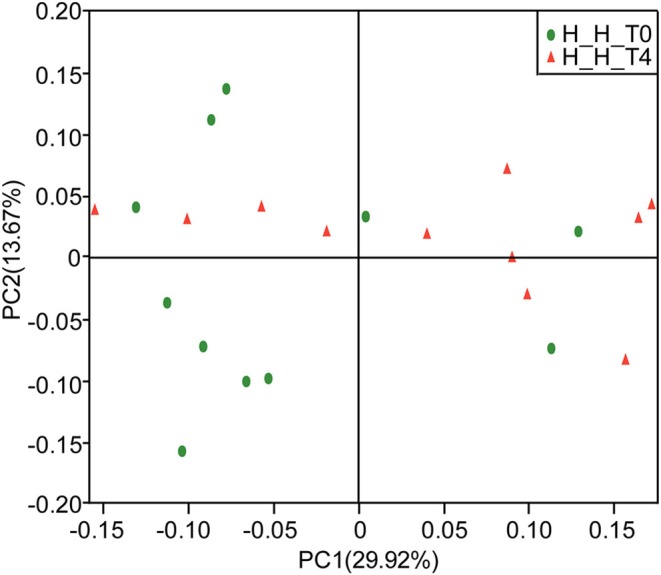
PCoA based on weighted UniFrac distances at the OTU level at 97% identity. Each sample is represented by a dot. Green balls represent the H-H-T0 samples. Red triangles represent the H-H-T4 samples. The samples did not form well-separated clusters corresponding to either group, suggesting that the microbial community structures are similar in the early and later stages of deciduous dentition.

PCoA based on the weighted UniFrac metric did not show distinct microbial profiles between the H-H-T0 and H-H-T4 groups (Figure 3), which was further confirmed by ANOSIM analysis (R = 0.0341, *P* = 0.234). These findings suggest that in healthy subjects, the salivary microbial diversity and community structure remain unchanged with age from the early to the later stage of deciduous dentition.

### Variations in microbiota composition related to caries

**Figure 4:**
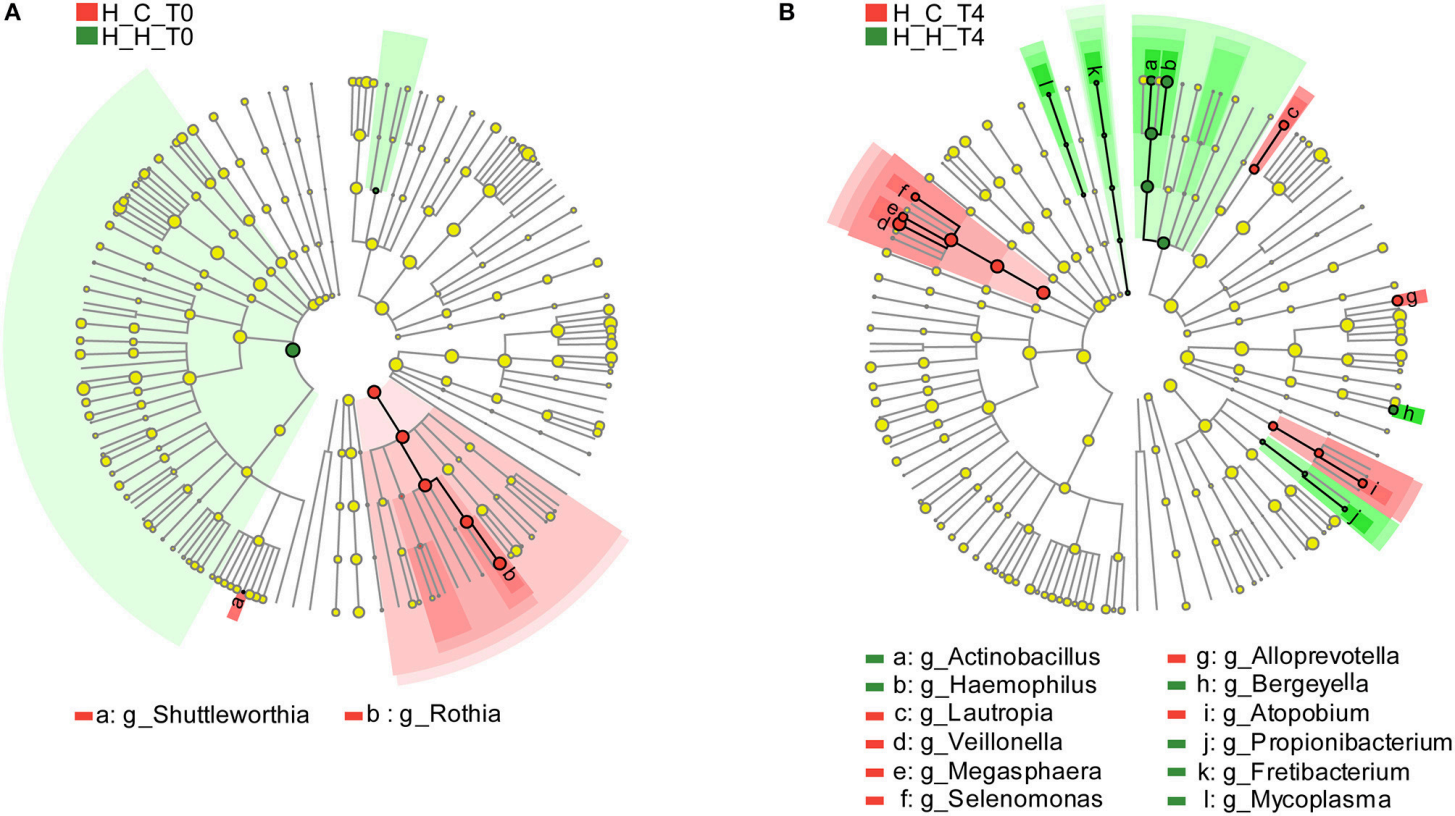
Comparison of microbial variations at the genus level, based on LEfSe analysis. Differences are represented by the color of the taxa (green indicating the H-H group, red indicating the H-C group). **(A)** Cladogram representing taxa with significantdifferences in abundance between the H-H-T0 and H-C-T0 groups. **(B)** Cladogram representing taxa with significant differences in abundance between the H-H-T4 and H-C-T4 groups.

As mentioned above, 127 genera were identified in the salivary samples. We examined variation in the microbial community composition associated with caries by comparing the relative abundance of these taxa between the H-H and H-C groups by LEfSe analysis. At baseline (T0), differences in microbial community composition were mainly due to the enrichment of Rothia and Shuttleworthia in the H-C-T0 group (Figure 4A). At the end of the trial (T4), the differences were attributable to the enrichment of *Actinobacillus, Bergeyella, Fretibacterium, Haemophilus, Mycoplasma*, and *Propionibacterium* in the H-H-T4 group and of *Alloprevotella, Atopobium, Lautropia, Megasphaera, Selenomonas*, and *Veillonella* in the H-C-T4 group (Figure 4B). Thus, the significant differences observed in the salivary abundance of *Alloprevotella, Atopobium, Lautropia, Megasphaera, Rothia, Selenomonas, Shuttleworthia*, and *Veillonella* between groups may be related to dental caries. To examine the possibility, we compared the H-H and H-C groups at the other three visits and found that there were three, five, and nine subjects with dental caries in the H-C group at T1, T2, and T3, respectively. At T1, the significant differences in microbial community composition were mainly due to the enrichment of *Bergeyella, Capnocytophaga, Haemophilus, Neisseria, Porphyromonas*, and *Streptococcus* in the H-H-T1 group and of *Atopobium, Megasphaera, Prevotella, Selenomonas*, and *Veillonella* in the H-C-T1 group (Figure S6A). At T2, the observed differences were mainly due to the enrichment of *Actinobacillus, Filifactor*, and *Peptococcus* in the H-H-T2 group and of *Dolosigranulum* in the H-C-T2 group (Figure S6B). At T3, *Eikenella* was significantly enriched in the H-H-T3 group, whereas *Atopobium, Megasphaera*, and *Veillonella* were enriched in the H-C-T3 group (Figure S6C). The fact that *Shuttleworthia* and *Rothia* were highly represented in the H-C group at baseline, but that no significant difference was found between the H-H and H-C groups during subsequent visits, indicates that the abundance of these two genera decreased significantly as caries occurred. In addition, the abundance of *Atopobium, Megasphaera*, and *Veillonella* did not differ between groups at baseline, but these genera were enriched in the H-C group at later visits except at T2, indicates that they increased significantly with the development of dental caries.

### Variations in microbiota composition related to age

**Figure 5:**
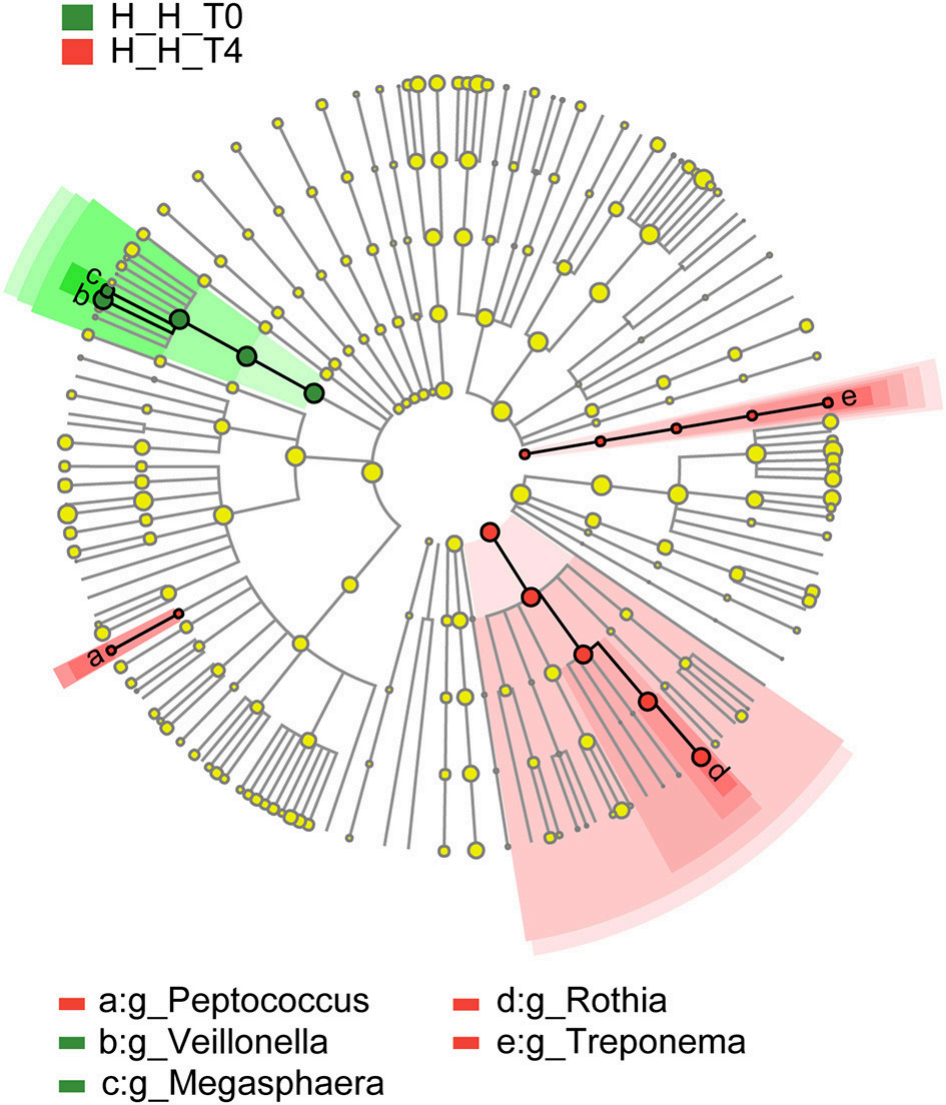
Comparison of microbial variations at the genus level, base on LEfSe analysis. Cladogram representing taxa with significant differences in abundance between the early and later stages of healthy deciduous dentition. Differences are represented by the color of the taxa (green indicating the H-H-T0 group, and red indicating the H-H-T4 group).

To assess variations in microbiota composition at different stages of healthy deciduous dentition, we compared the H-H-T0 and H-H-T4 groups by LEfSe analysis. We found that *Megasphaera* and *Veillonella* were enriched in the H-H-T0 group, whereas *Peptococcus, Rothia*, and *Treponema* were enriched in the H-H-T4 group (Figure 5). These findings revealed that significant variations in salivary microbiota composition occurred with age, even during the deciduous dentition period.

### Composition and variations in salivary core microbiome in preschool children

**Figure 6:**
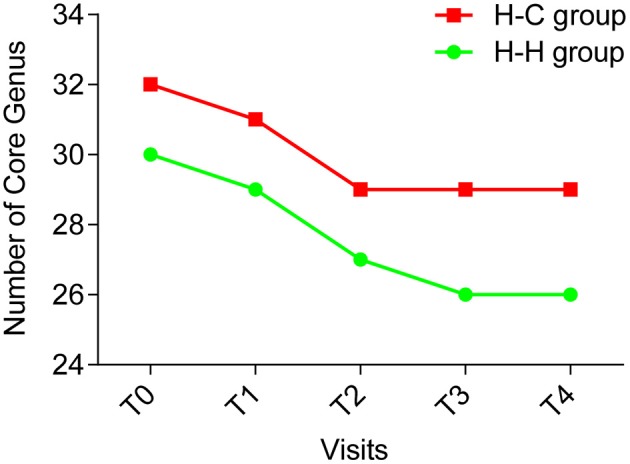
The number of core genera at each time point (green line indicating the H-H group, red line indicating the H-C group). The number of core genera decreased during the first 4 time points and became stable (26 core genera) in the H-H group at the last time point; and the number of the core genera decreased during the first 3 time points and became stable (29 core genera) in the H-C group for the remaining 2 time points.

The core microbiome is defined as a set of microbial taxa shared by all subjects (Turnbaugh et al., 2007), and a more strict definition is “common” core microbiome which refers to the microbial taxa present in the same subjects at all time points (Lazarevic et al., 2010). We examined the core microbiome of preschool children in the H-H and H-C groups at the genus level, and found that the number of core genera decreased over time (Figure 6). In the H-H group, 30, 29, and 27 core genera were detected at T0, T1, and T2, respectively. At T3 and T4, 26 core genera were identified. In the H-C group, 32, and 31 core genera were found at T0 and T1, respectively, while 29 core genera were detected at T2, T3, and T4. These results underscore the importance of sampling at several time points (at least four) in order to define a core microbiome.

**Figure 7:**
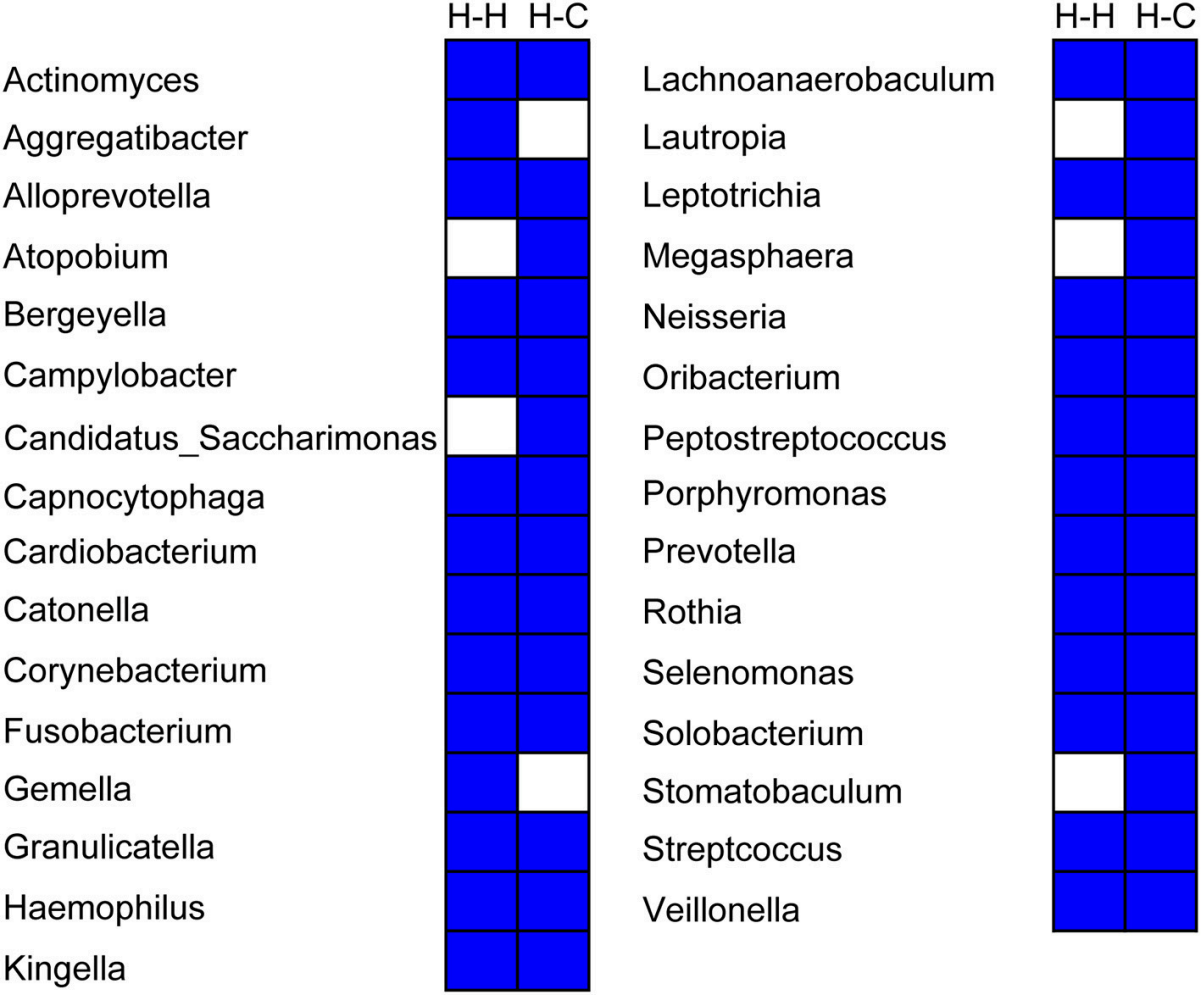
Composition of the core microbiome in the H-H and H-C groups. Each column represents a defined group. For each column, the blue cells indicate that the genus listed on the left had fallen into the “core microbiome” of this group, and the white cells indicate that the genus listed on the left could not be observed in all samples of this group.

Twenty-six genera were detected in all samples in the H-H group that accounted for 94.2% of total sequences, whereas 29 genera accounting for 94.0% of all sequences were found in the H-C group samples. Thus, the core microbiome represents a significant proportion of total oral microbes in preschool children, irrespective of the occurrence of dental caries. Twenty-four genera were common to the H-H and H-C groups (Figure 7), indicating that the core microbiomes of both groups were highly similar despite some differences, including relative abundance of *Bergeyella, Capnocytophaga, Haemophilus, Kingella, Neisseria, Porphyromonas, Prevotella, Rothia, Selenomonas, Streptococcus*, and *Veillonella* at the predetermined time points (Figure S7). For instance, *Bergeyella* was more prevalent in the H-H group than in the H-C group at T1 and T4.

## Discussion

The oral cavity is one of the most diverse microbial habitats in the human body, harboring around 1,000 bacterial species (Wade, 2013). About half of all oral bacteria are uncultivable, posing a challenge for studies on the oral microbiome. Culture-independent methods such as high-throughput or next-generation sequencing can overcome this problem (Ye et al., 2012). The Illumina MiSeq platform is superior to older technologies such as Roche/454 pyrosequencing since it provides a similar read length at a fraction of the cost (Fadrosh et al., 2014). Different hypervariable regions (V1-V9) of bacterial 16S rRNA gene can be analyzed for accuracy and reliability; it was previously found that the V3-V4 and V4-V5 regions yielded the most accurate results, regardless of sequencing technology and quality (Claesson et al., 2010). We therefore analyzed the V3-V4 regions in salivary samples by Illumina MiSeq sequencing.

Unweighted UniFrac analysis is a qualitative measure of the community β diversity, and disregarding the relative abundance of microbes can better detect the effects of different founding populations. On the contrary, weighted UniFrac is a quantitative measure of the community β diversity, which can detect changes in how many sequences from each lineage are present, as well as detect changes in which taxa are present (Chang et al., 2011). In view of the relative abundance of different kinds of bacteria can be critical for describing community changes; thus, it was more suitable to use weighted UniFrac in this study. We utilized a combination of weighted UniFrac and ANOSIM to assess differences between microbial communities to obtain more reliable results.

Previous data have shown that the oral microbial community composition varies according to the dentition status (Lif Holgerson et al., 2015; Xu et al., 2015). The mouth is the part of the digestive system that is in contact with the external environment; as such, oral microbial communities are highly dynamic (Parahitiyawa et al., 2010). We speculated that the residence time in the oral cavity may affect the microbial composition, and changes over the course of a given dentition stage. We analyzed the salivary microbiota composition in children with healthy deciduous dentition from the age of 4 to 6 years and observed age-related changes in microbial community, which supports our hypothesis. So far as we know, this is the first study to explore changes in salivary microbiota from the early to later stages of deciduous dentition. The results suggested that age affceted the saliva microbial community composition during the period of deciduous dentition, which reminds us that it is necessary to consider the influence of age fully when detecting the salivary microbiota, even in the same dentition period.

In our study, we found that *Megasphaera* and *Veillonella* were enriched in the H-H-T0 group, whereas *Peptococcus, Rothia*, and *Treponema* were enriched in the H-H-T4 group when comparing these two groups. Combining our study with previous studies (Ling et al., 2010; Jiang et al., 2014; Xiao et al., 2016), we also found that *Megasphaera* and *Veillonella* were associated with dental caries, whereas *Peptococcus, Rothia*, and *Treponema* were related to dental health., These findings suggest that the salivary environment might improve as children age from 4 to 6 years old, thus lowering the risk of dental caries.

Oral microbes prevent the colonization of extrinsic bacteria, thereby regulating systemic health. On the other hand, their perturbation can lead to oral diseases such as dental caries (Shimada et al., 2015) and periodontitis (Kirst et al., 2015). In the study, we identified changes in the microbial community composition and structure related to dental caries, which provides information regarding microbial variations of dental caries and may be important for its early diagnosis and prevention. We conducted a follow-up case-control study and expected to eliminate potential biases associated with age, gender, and sample selection to make our results more precise than those of cross-sectional studies. Indeed, we found inter-group differences at the beginning of the study and significant microbial variations related to age, even during the same dentition status. These differences were not considered adequately in previous cross-sectional studies, which serves as an important reminder that follow-up case-control studies may be more suitable for researching microbial variations and can provide more reliable results.

It has been reported that oral microbial diversity decreases with the occurrence of caries (Gross et al., 2010; Jiang et al., 2014). However, microbial diversity did not differ between children with and without dental caries in our study. This may be due to the homogeneity of the study population: all subjects were from the same geographical region and had a similar diet and lifestyle. Thus, although differences in microbial composition were seen, both groups of subjects exhibited similar microbial diversity, which is consistent with a previous study in which microbial diversity was found to be similar between twin children with discordant caries phenotypes (Zhang et al., 2015).

Compared to other microbial habitats in the human body, such as the gut, the oral cavity is characterized as a complex habitat because of the presence of both soft and hard tissues (Qiao et al., 2018). Microbes colonize at different oral niches, and these organisms differ markedly in terms of community structure and composition. Salivary specimens are easier to collect compared to other sources (such as dental plaque, mucosa) from the preschool children. Moreover, salivary specimens provide a different perspective from previous studies that mainly focused on dental plaque. Therefore, we analyzed saliva samples, anticipating that there would be public interest in salivary sampling-based research and clinical application, such as early diagnosis and prevention of dental caries, especially in young children.

In our study, *Atopobium, Megasphaera*, and *Veillonella* were more abundant in subjects with dental caries. This finding is in agreement with earlier studies demonstrating that *Atopobium* and *Megasphaera* were more prevalent on cavitated dentine lesions than on healthy teeth (Jiang et al., 2014) and that *Atopobium* was associated with caries in children at 3 years of age (Lif Holgerson et al., 2015). A next-generation sequencing analysis of the bacterial composition of dentine caries in a Japanese population revealed a predominance of *Atopobium* (Obata et al., 2014). In addition, *Veillonella* was abundant in a high-caries population (Xiao et al., 2016). *Veillonella* in dental plaque was found to be associated with the development of caries (Ling et al., 2010); it was more abundant in the saliva of caries-active subjects, as compared to healthy subjects (Li et al., 2016) and in the plaque of subjects with caries along with cariogenic *Streptococcus* species (Chalmers et al., 2008), and was shown to play a key role in the development of dental plaque (Periasamy and Kolenbrander, 2010). These findings suggest that overrepresentation of *Veillonella* in saliva is an indicator of increased risk for dental caries in deciduous dentition. A significant finding of our study is that a diversity of genera was observed in saliva samples from preschool children, in contrast to plaque or dentine caries samples in children or adults examined in other studies.

We found that *Shuttleworthia* and *Rothia* decreased significantly with the development of dental caries. *Shuttleworthia satelles* was previously shown to be more abundant in the saliva of children without dental caries, as compared to those with dental caries (ElSalhy et al., 2016). Accordingly, *Shuttleworthia satelles* was the sole member of genus *Shuttleworthia* detected in our study. On the contrary, *Shuttleworthia* was found to be more abundant in supragingival plaque from cavitated dentine lesions than that from healthy teeth (Jiang et al., 2014). These results are inconsistent, and the source of samples was different between these two studies. Thus wespeculated that the use of different sample types (saliva vs. supragingival plaque) may explain why contradictory results were found. Although previous studies demonstrated that *Rothia* is prevalent in saliva and plaque samples in subjects with caries (Jagathrakshakan et al., 2015; Xiao et al., 2016), no statistical difference was found in the relative abundance of *Rothia* between subjects with caries and those without caries. In contrast, we found that the prevalence of *Rothia* was reduced significantly in caries-affected subjects, indicating that this genus is associated with dental health.

*Streptococcus* plays a well-documented role in dental caries (Milnes and Bowden, 1985; Van Houte et al., 1990). For instance, *S. mutans* is a well-known cariogenic species due to its high capacity for acid production and acid tolerance (Loesche, 1986). However, we did not observe any statistical difference in *Streptococcus* abundance between the H-H and H-C groups in this study, in accordance with the findings of others (Ling et al., 2010; Xu et al., 2014; Jiang et al., 2016). A possible explanation for this is that *Streptococcus* comprises many species including *S. mutans, S. sanguinis, S. oralis, S. sobrinus, S. mitis*, and so on that may play distinct roles—and hence, exhibit variable abundance—during the development of dental caries. For instance, *S. dentisani*, a new streptococcus species isolated from dental plaque of caries-free individuals, was proposed to be positive in the oral cavity. It does not produce any toxic secondary metabolites. Furthermore, this species inhibits the growth of pathogens and also buffers acidic pH (an important cause of dental caries) (López-López et al., 2017). However, additional studies are needed to evaluate this possibility.The core microbiome of the healthy oral cavity has been defined using next generation sequencing (Zaura et al., 2009); however, it is known to change according to age and niche (i.e., saliva, plaques, and mucosa) (Xu et al., 2015). Furthermore, the oral core microbiome was found be dynamic during head-and-neck radiotherapy (Hu et al., 2013), and may be influenced by various conditions, including the sample source, dentition stage, and disease status. In this study, we adopted a strict definition of the core microbiome, which is that it is present in all subjects regardless of the abundance (Huse et al., 2012). The core microbiomes of the H-H and H-C groups comprised different taxa, although many others were common to both; 11 of the shared genera showed different relative abundances between both groups, providing evidence that the core microbiome changes with the development of caries. Therefore, different sub-populations of the core microbiome should be defined in the context of pathophysiological conditions. Given that core microbiomes also vary according to the oral niche (Xu et al., 2015), the relationship between the oral core microbiome and caries merits a more detailed analysis.

A limitation of this study is that, due to the constraints of the sampled material and potential risks, no radiographs were obtained to detect dental caries. Therefore, some small lesions on the proximal tooth surface may have been overlooked during our visual and tactile inspections. To minimize error, we assessed dental health using the ICDAS system, which has proven validity and sensitivity (Pitts, 2004; Cadavid et al., 2010). Furthermore, due to the large number of subjects who were lost to follow-up, our sample size was small compared to that of previous studies (Ling et al., 2010; Yang et al., 2012), which may have influenced the microbial diversity to some extent. Our study was focused on preschool children with complete deciduous dentition; hence, the span of follow-up was limited to 2 years, which may not be long enough. Subjects with eruption of permanent teeth were excluded during these 2 years, which partly led to the small sample size in this study. These above limitations restricted our conclusions to a specific context and need to be eliminated in future studies.

In conclusion, we found that, even during the deciduous dentition period, age-related differences occurred in the microbial community composition. Our longitudinal follow-up, case-control study eliminated potential biases associated with age, gender, and sample selection, making our results more accurate than those of cross-sectional studies. Our data showed that salivary microbial structure and composition changed significantly with the development of dental caries. These findings improve our understanding of variations in the salivary microbiome with dental caries and age during deciduous dentition, which may be useful for identifying children who are at risk for tooth decay.

## Author contributions

HC, XC, and LX contributed conception and design of the study; LX, XC, YW, WJ, and SW performed the experiments; LX, WJ, ZL, and HC performed the data analysis; LX wrote the manuscript; LX, ZL, and HC revised the manuscript. All authors read and approved the submitted version.

### Conflict of interest statement

The authors declare that the research was conducted in the absence of any commercial or financial relationships that could be construed as a potential conflict of interest.
