# Supplementary figures and images for: Dynamic Alterations in Salivary Microbiota Related to Dental Caries and Age in Preschool Children With Deciduous Dentition: A 2-Year Follow-Up Study
Source: Front Physiol. 2018 Apr 4;9:342. doi: 10.3389/fphys.2018.00342 (PMC5893825; doi:10.3389/fphys.2018.00342)

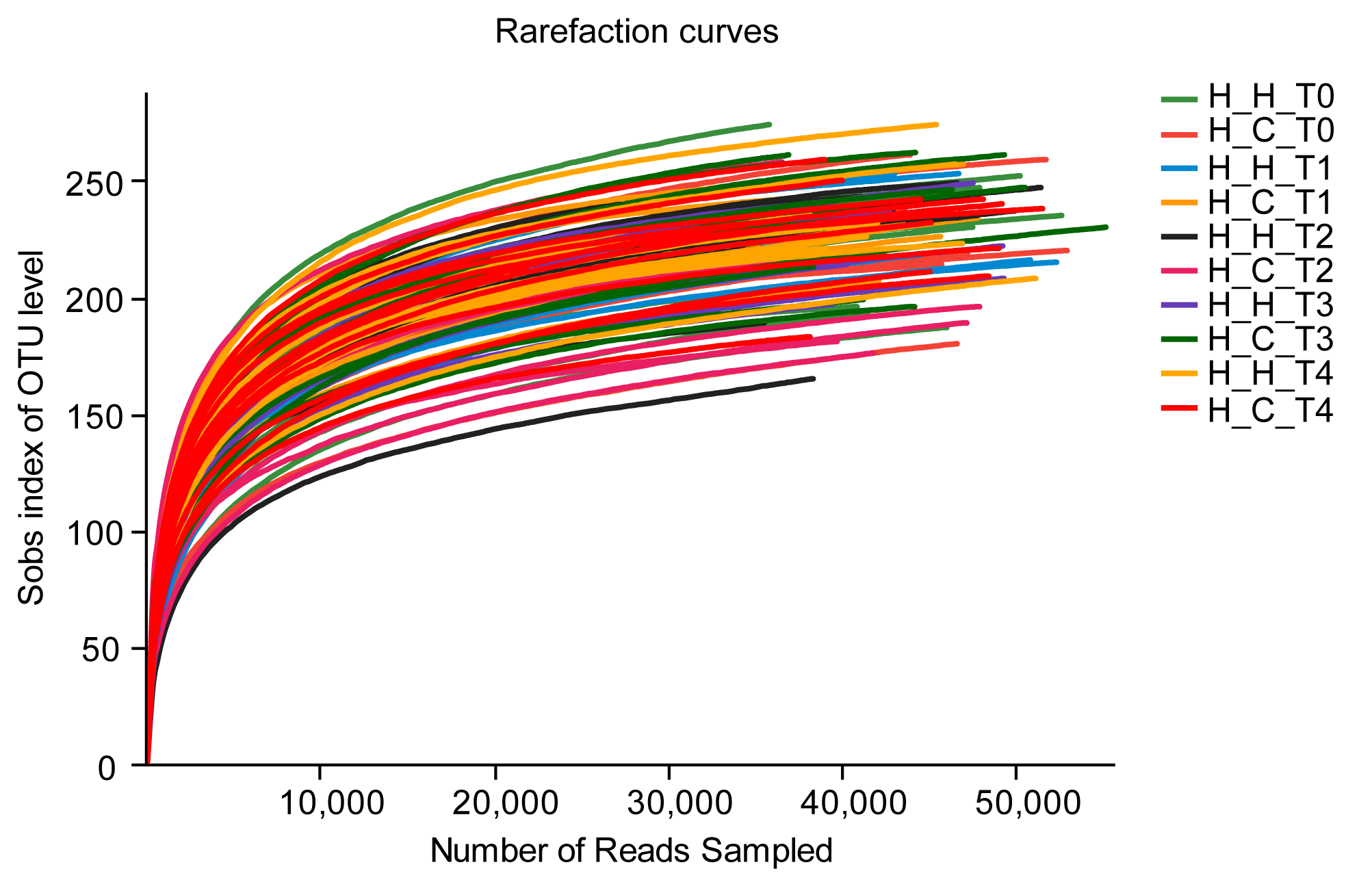

Supplement: Supplementary file 1 [file Image1.TIF]

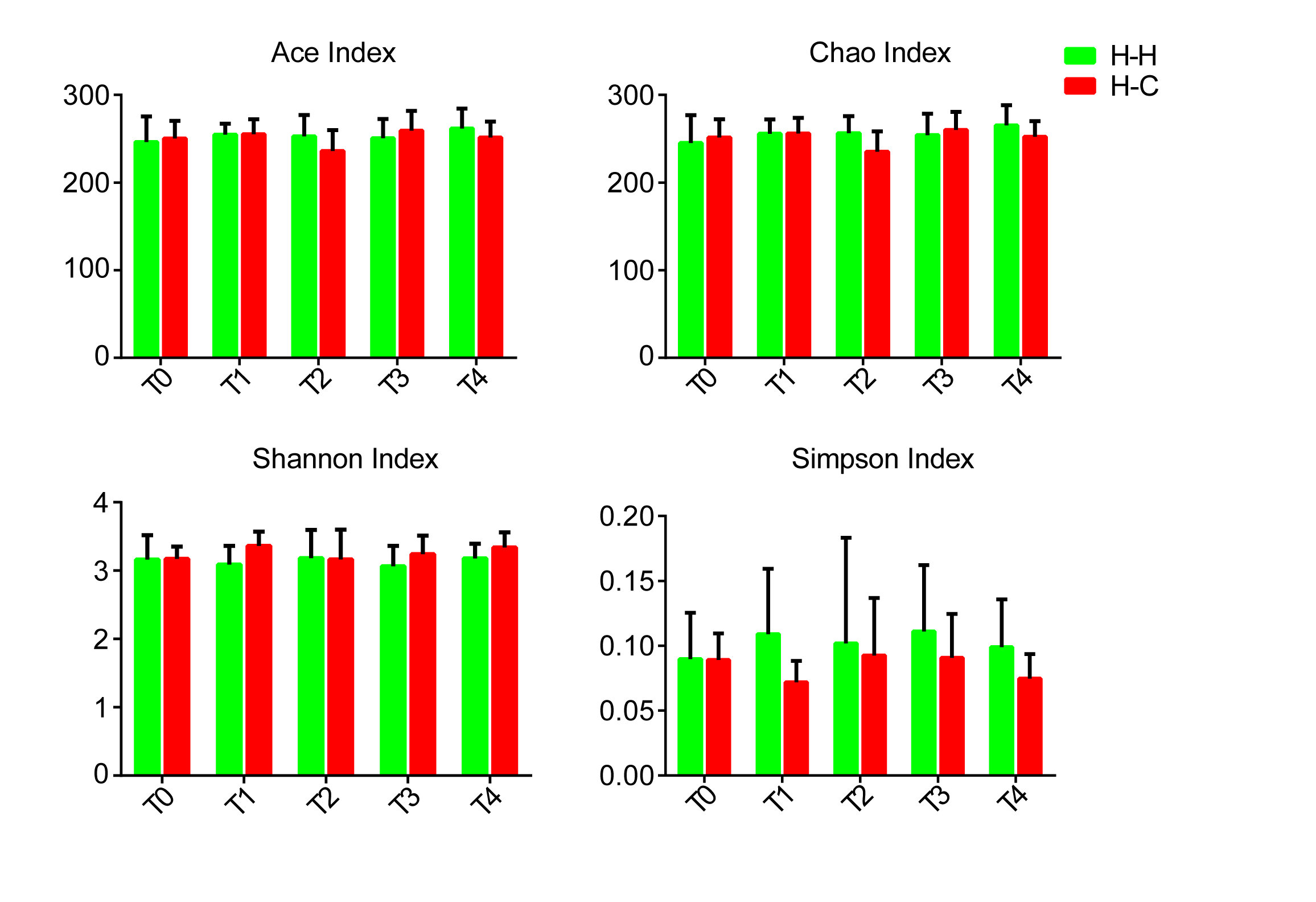

Supplement: Supplementary file 2 [file Image2.TIF]

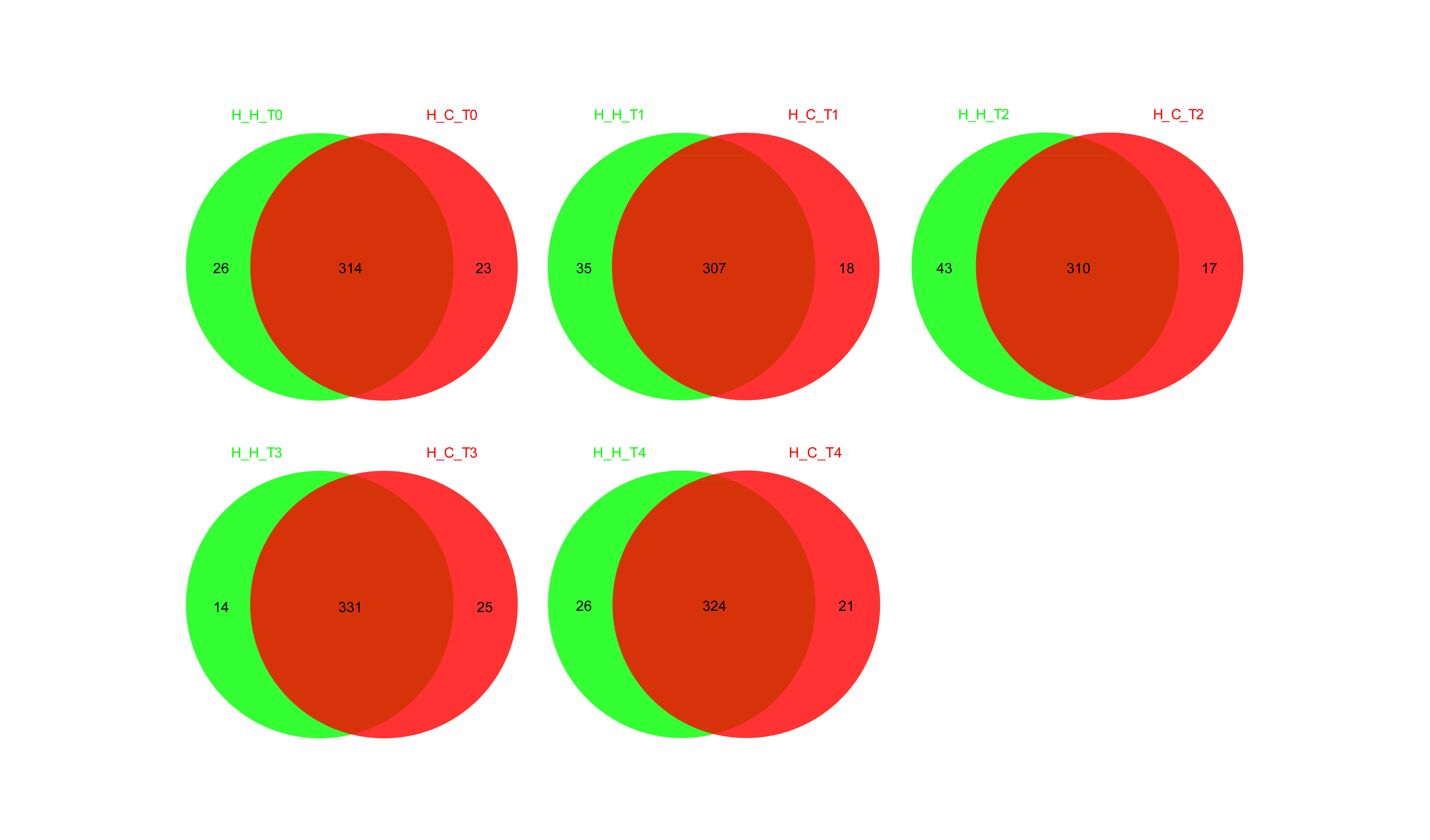

Supplement: Supplementary file 3 [file Image3.TIF]

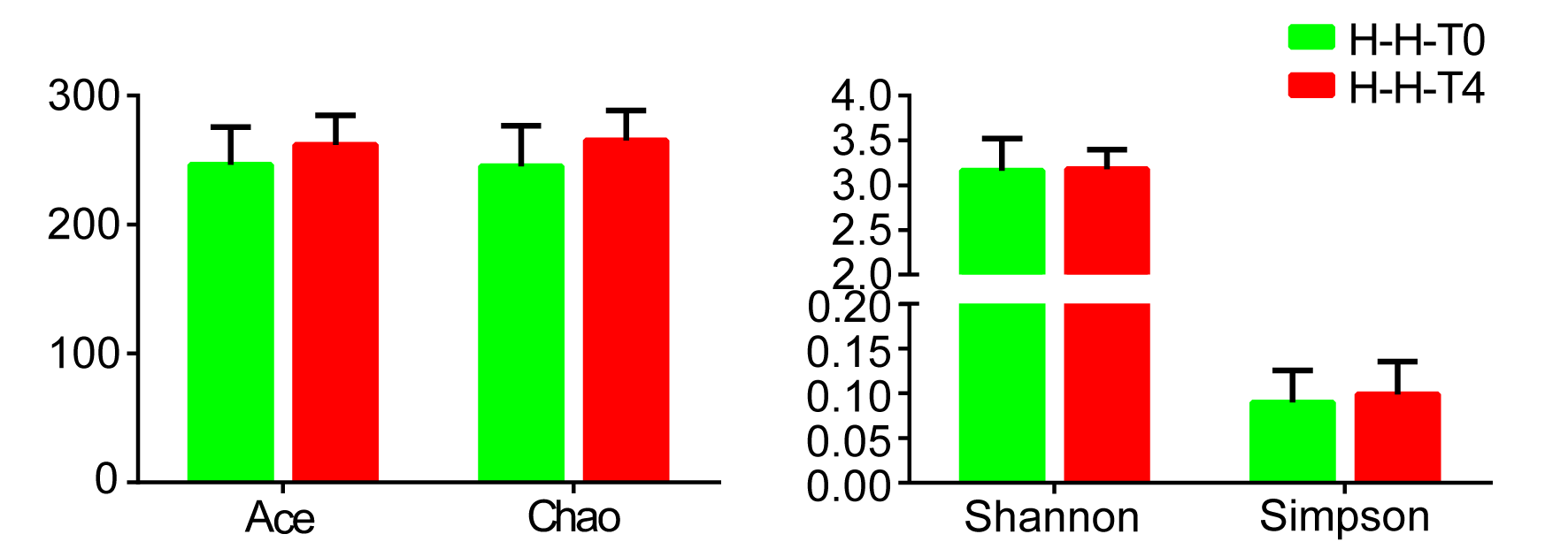

Supplement: Supplementary file 4 [file Image4.TIF]

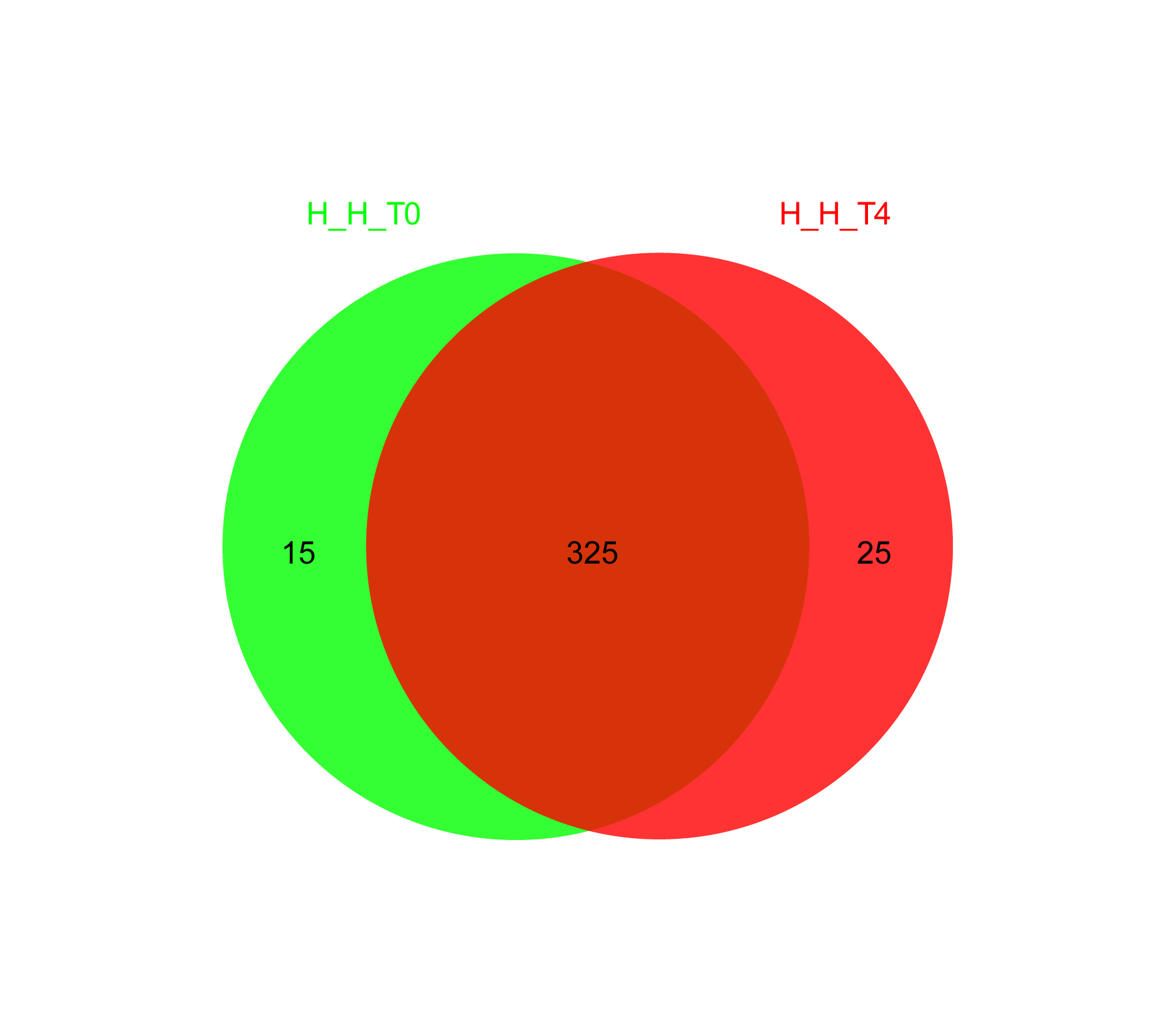

Supplement: Supplementary file 5 [file Image5.TIF]

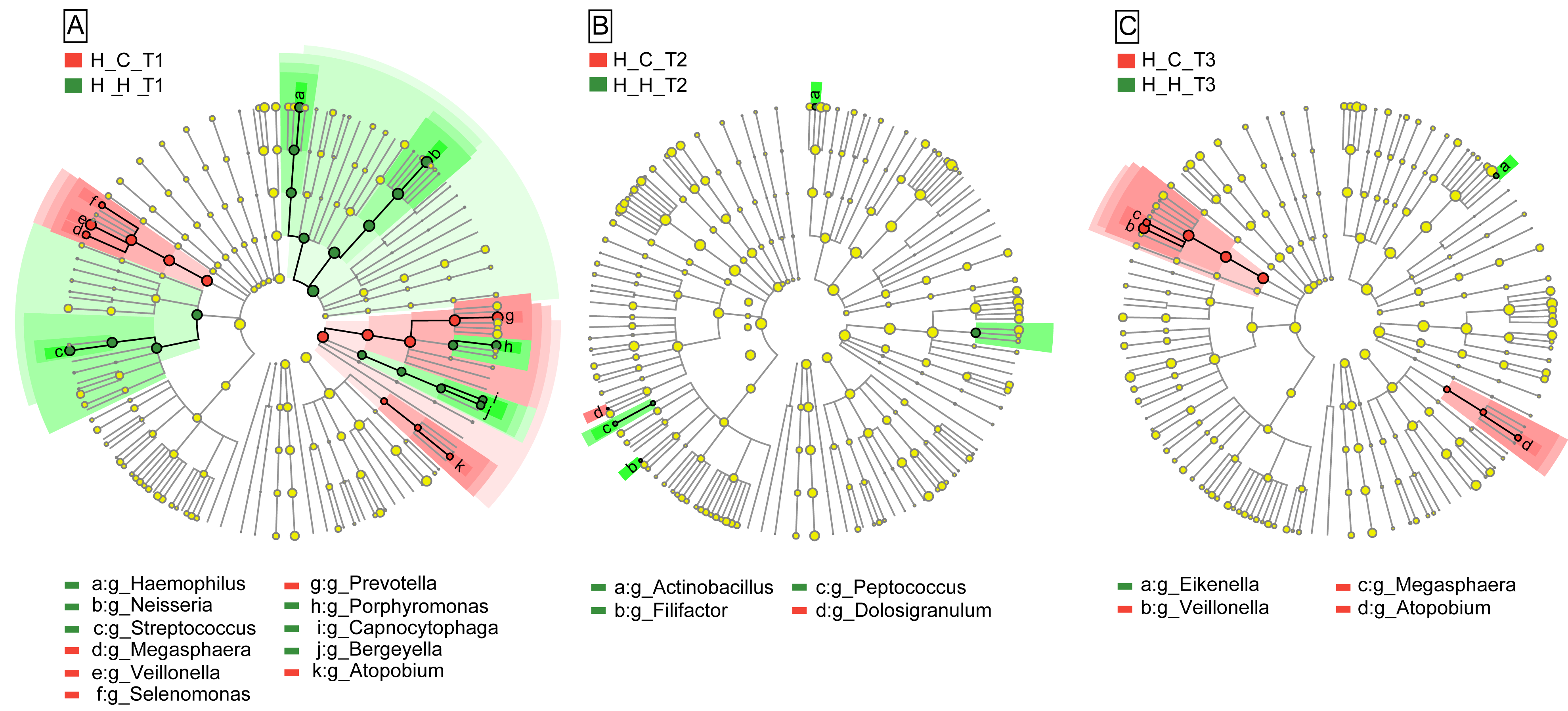

Supplement: Supplementary file 6 [file Image6.TIF]

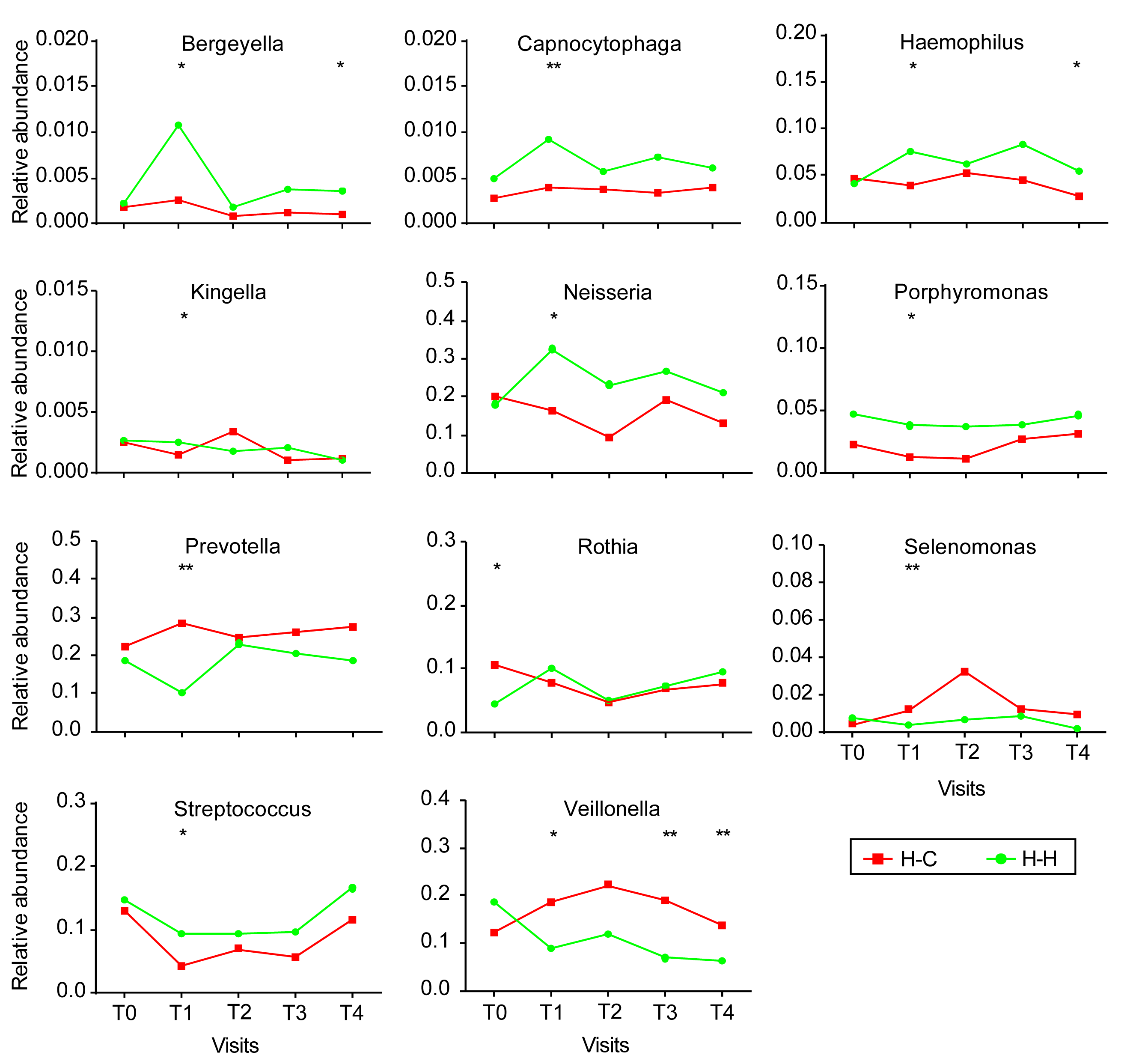

Supplement: Supplementary file 7 [file Image7.TIF]
