# Supplementary material for: Dynamic Alterations in Salivary Microbiota Related to Dental Caries and Age in Preschool Children With Deciduous Dentition: A 2-Year Follow-Up Study
Source: Front Physiol. 2018 Apr 4;9:342. doi: 10.3389/fphys.2018.00342 (PMC5893825; doi:10.3389/fphys.2018.00342)
Supplement: Supplementary file 8 [file Presentation1.PDF]

FIGURE S1 Rarefaction curves are used to estimate richness among the 10 groups. The ordinate shows the number of OTUs that would be expected to be found after sampling the number of sequences shown on the abscissa.

FIGURE S2 Comparing of alpha diversity indices (Ace, Chao, Shannon, and Simpson) between H-H and H-C groups at five time points (T0, T1, T2, T3, and T4). Data are shown as mean and SD, and there is no statistical difference between H-H and H-C groups by non-parametric Mann-Whitney U test.

FIGURE S3 Venn diagrams show the overlapping of all OTUs, which were calculated at the 97% similarity level, between H-H and H-C groups at five time points. A high degree of similarities around 90% presented between groups. For example, 324 OTUs are shared by the H-H-T4 and H-C-T4 groups, accounting for 92.6% and 93.9% of their total OTUs, respectively.

FIGURE S4 Comparing of alpha diversity indices (Ace, Chao, Shannon, and Simpson) between H-H-T0 and H-H-T4 groups. Data are shown as mean and SD, and there is no statistical difference by nonparametric Mann-Whitney U test.

FIGURE S5 Venn diagrams show the overlapping of all OTUs, which were calculated at the 97% similarity level, between H-H-T0 and H-H-T4 groups. A high degree of similarities presented between groups, 325 OTUs are shared by the H-H-T0 and H-H-T4 groups, accounting for 95.6% and 92.9% of their total OTUs, respectively.

FIGURE S6 Comparison of microbial variations at the genus level, based on LEfSe analysis. Differences are represented by the color of the taxa (green indicating the H-H group, and red indicating the H-C group). (A) Cladogram representing taxa with significant differences in abundance between H-H-T1 and H-C-T1 groups. (B) Cladogram representing taxa with significant differences in abundance between H-H-T2 and H-C-T2 groups. (C) Cladogram representing taxa with significant differences in abundance between H-H-T3 and H-C-T3 groups.

FIGURE S7 Temporal variations in relative abundance of shared 11 common core genera. The abscissas and ordinates represent five time points and relative abundance of each genus respectively (different in ordinate scale). The green lines represent H-H group, and the red lines represent H-C group. All data are shown as mean and SD, and the statistical differences are marked by asterisks (*P < 0.05, **P < 0.01 by non-parametric Mann-Whitney U test).
