# Supplementary material for: Dynamic Alterations in Salivary Microbiota Related to Dental Caries and Age in Preschool Children With Deciduous Dentition: A 2-Year Follow-Up Study
Source: Front Physiol. 2018 Apr 4;9:342. doi: 10.3389/fphys.2018.00342 (PMC5893825; doi:10.3389/fphys.2018.00342)
Supplement: Supplementary file 9 [file Presentation2.PDF]

Informed consent

Background and objective:

Dental caries is the most common oral disease in children, and its incidence is over 60% in China. Lowering its occurrence is crucial for reducing the burden to families and society. Thus, it is important to determine the etiology and thus provide effective preventive measures. Dental caries is closely related to oral microbes, and we are conducting a study developed by Professor Chen Hui and the Affiliated Hospital of Stomatology, Medical College, Zhejiang University, in order to reveal the relationship between them. You are invited to participate in this study to help us to achieve the research purpose. The study was reviewed and approved of by the ethics committee of the Affiliated Hospital of Stomatology, Medical College, Zhejiang University.

Exclusion criteria:

1. The use of antibiotics, probiotics, synbiotics, or fluoride within the prior 3 months
2. Apparent active bacterial or viral infection in any part of the body
3. Visually detectable enamel or dentin hypoplasia
4. Eruption of permanent teeth during the study
5. Any dental caries with or without restoration

Methods:

Sixty subjects will be recruited for this study, and they will be examined and sampled at five time points: at the beginning of the study and at 6, 12, 18, and 24 months after the study has begun. Sampling will be performed in the morning before brushing, gargling, and breakfast. Two milliliters of spontaneous, non-stimulated whole saliva will be sampled at each time point. The subjects' caries status will be determined by oral clinical examination according to criteria from the International Caries Detection and Assessment System.

Benefits:

We expect to identify oral diseases in a timely manner, and we can provide advice and necessary treatments for you if needed. We will prioritize your appointments if you want to treat your oral disease, and registration is free. The results of this research will provide information regarding the relationship between dental caries and salivary microbes.

Risk and discomfort:

There is a risk for pain and injury during oral clinical examination and obtaining samples from the mouth. You may experience hunger and bad breath on the mornings that samples are taken. We will do our best to obtain samples early in the morning to alleviate or avoid these issues.

Fees and compensation:

We will not charge any fees for participating in the study. You will get free treatment and appropriate compensation for any injury during the sampling.

Privacy:

During the processes of collecting, storing, and using samples, they will be uniformly numbered and your personal information will be kept strictly confidential. Information that can reveal your identity will not be disclosed to members other than the research team. We will make every effort to protect the privacy of your medical data within the bounds of the law. The samples and your information will be saved in the Affiliated Hospital of Stomatology, Medical College, Zhejiang University. The government administrative department or the ethic committee members will have access to your information in accordance with provisions of personal data, when necessary. The samples will only be used for research purposes. Your identity will not be disclosed in any research reports.

Communication:

If you have any questions related to this study, including the participant's rights and interests, or if you experience any discomfort or injury during the study, please contact us at 0571-87217437. Please provide your contact number and address so that we can inform you in a timely manner if any new information pertinent to this study becomes available.

Voluntary participation:

Whether to take part in this study is entirely up to you. You can refuse to participate, or withdraw during the course of the study. It will not hurt our relationship with you or affect your medical interests. If you are willing to participate in this study, please provide us with an accurate medical history and physical condition. Please do not take restricted drugs. Please inform the researchers if you have been involved in any other clinical studies. Out of consideration for your interests, we may discontinue the study if necessary, and your data will not be included in the results of the study. We hope that you can complete the two-year follow-up study.

Statement of consent

All my questions have been answered and I am fully aware of the parameters and purpose of the study. I well understand the risks and benefits of participating in this study. I can refuse or withdraw at any time during the study. I will provide the researchers accurate information regarding my physical condition. I will contact the researchers if I need take any restricted medicine. I agree to take part in this study voluntarily, and I will receive a copy of the written informed consent form.

Participant signature: **Date:**

Phone number:

Guardian signature: **Date:**

Phone number:

Researcher signature: **Date:**

Phone number:
