# Supplementary material for: Dynamic Alterations in Salivary Microbiota Related to Dental Caries and Age in Preschool Children With Deciduous Dentition: A 2-Year Follow-Up Study
Source: Front Physiol. 2018 Apr 4;9:342. doi: 10.3389/fphys.2018.00342 (PMC5893825; doi:10.3389/fphys.2018.00342)
Supplement: Supplementary file 10 [file Table1.DOCX]

Table S1 The number of samples per group and the number of subjects with caries and dfs per visit.

| Visit | Total | H-H | H-C | Subjects with caries dfs index |
| --- | --- | --- | --- | --- |
| T0 | 23 | 11 | 12 | 0 0 |
| T1 | 17 | 9 | 8 | 3 6 |
| T2 | 18 | 11 | 7 | 5 15 |
| T3 | 19 | 9 | 10 | 9 33 |
| T4 | 23 | 11 | 12 | 12 58 |
