# Supplementary material for: Dynamic Alterations in Salivary Microbiota Related to Dental Caries and Age in Preschool Children With Deciduous Dentition: A 2-Year Follow-Up Study
Source: Front Physiol. 2018 Apr 4;9:342. doi: 10.3389/fphys.2018.00342 (PMC5893825; doi:10.3389/fphys.2018.00342)
Supplement: Supplementary file 11 [file Table2.DOCX]

Table S2 Distribution of the dominant microbes in different groups at phylum level (relative abundance, Mean ± SD).

| Group | Actinobacteria  Mean SD | Bacteroidetes  Mean SD | Candidate_division_TM7  Mean SD | Firmicutes  Mean SD | Fusobacteria  Mean SD | Proteobacteria  Mean SD |
| --- | --- | --- | --- | --- | --- | --- |
| H-H-T0 | 0.063±0.061 | 0.257±0.108 | 0.026±0.031 | 0.393±0.105 | 0.021±0.013 | 0.238±0.159 |
| H-C-T0 | 0.134±0.085 | 0.256±0.169 | 0.018±0.017 | 0.301±0.100 | 0.022±0.019 | 0.265±0.180 |
| H-H-T1 | 0.117±0.051 | 0.168±0.075 | 0.011±0.007 | 0.229±0.097 | 0.039±0.025 | 0.433±0.137 |
| H-C-T1 | 0.094±0.081 | 0.321±0.130 | 0.028±0.019 | 0.279±0.074 | 0.059±0.022 | 0.219±0.123 |
| H-H-T2 | 0.064±0.034 | 0.295±0.140 | 0.023±0.024 | 0.266±0.109 | 0.034±0.021 | 0.316±0.198 |
| H-C-T2 | 0.062±0.045 | 0.285±0.133 | 0.060±0.060 | 0.374±0.152 | 0.049±0.049 | 0.168±0.146 |
| H-H-T3 | 0.083±0.059 | 0.267±0.134 | 0.027±0.026 | 0.219±0.085 | 0.027±0.015 | 0.371±0.149 |
| H-C-T3 | 0.081±0.037 | 0.312±0.146 | 0.026±0.023 | 0.295±0.110 | 0.028±0.019 | 0.254±0.152 |
| H-H-T4 | 0.121±0.080 | 0.250±0.144 | 0.016±0.017 | 0.293±0.080 | 0.040±0.021 | 0.278±0.148 |
| H-C-T4 | 0.105±0.055 | 0.323±0.098 | 0.029±0.018 | 0.318±0.062 | 0.049±0.027 | 0.170±0.098 |
| H-H-ALL | 0.089±0.062 | 0.250±0.126 | 0.021±0.023 | 0.284±0.112 | 0.032±0.020 | 0.321±0.169 |
| H-C-ALL | 0.099±0.066 | 0.299±0.135 | 0.030±0.030 | 0.311±0.100 | 0.040±0.030 | 0.218±0.143 |
