# Supplementary material for: Dynamic Alterations in Salivary Microbiota Related to Dental Caries and Age in Preschool Children With Deciduous Dentition: A 2-Year Follow-Up Study
Source: Front Physiol. 2018 Apr 4;9:342. doi: 10.3389/fphys.2018.00342 (PMC5893825; doi:10.3389/fphys.2018.00342)
Supplement: Supplementary file 12 [file Table3.DOCX]

Table S3 Distribution of the dominant microbes in different groups at genus level (relative abundance, Mean ± SD).

| Group | Actinomyces  Mean SD | Alloprevotella  Mean SD | | Fusobacterium  Mean SD | | Haemophilus  Mean SD | | Leptotrichia  Mean SD | | Neisseria  Mean SD |
| --- | --- | --- | --- | --- | --- | --- | --- | --- | --- | --- |
| H-H-T0 | 0.016±0.015 | 0.015±0.026 | | 0.009±0.007 | | 0.042±0.019 | | 0.010±0.006 | | 0.180±0.139 |
| H-C-T0 | 0.025±0.025 | 0.008±0.005 | | 0.009±0.014 | | 0.047±0.035 | | 0.013±0.010 | | 0.203±0.154 |
| H-H-T1 | 0.014±0.023 | 0.008±0.006 | | 0.016±0.009 | | 0.075±0.041 | | 0.022±0.017 | | 0.326±0.123 |
| H-C-T1 | 0.012±0.008 | 0.016±0.014 | | 0.011±0.011 | | 0.039±0.029 | | 0.046±0.026 | | 0.162±0.102 |
| H-H-T2 | 0.012±0.008 | 0.019±0.015 | | 0.021±0.018 | | 0.062±0.051 | | 0.012±0.009 | | 0.231±0.187 |
| H-C-T2 | 0.013±0.011 | 0.022±0.013 | | 0.010±0.009 | | 0.054±0.068 | | 0.039±0.048 | | 0.093±0.097 |
| H-H-T3 | 0.008±0.008 | 0.013±0.009 | | 0.016±0.011 | | 0.084±0.068 | | 0.011±0.007 | | 0.267±0.135 |
| H-C-T3 | 0.011±0.006 | 0.020±0.010 | | 0.010±0.009 | | 0.045±0.030 | | 0.017±0.012 | | 0.191±0.142 |
| H-H-T4 | 0.024±0.022 | 0.007±0.005 | | 0.020±0.017 | | 0.054±0.037 | | 0.019±0.017 | | 0.210±0.126 |
| H-C-T4 | 0.026±0.033 | 0.012±0.010 | | 0.022±0.030 | | 0.028±0.022 | | 0.027±0.022 | | 0.130±0.091 |
| H-H-ALL | 0.015±0.017 | 0.012±0.015 | | 0.017±0.014 | | 0.062±0.046 | | 0.015±0.013 | | 0.239±0.148 |
| H-C-ALL | 0.018±0.022 | 0.015±0.011 | | 0.013±0.018 | | 0.042±0.037 | | 0.026±0.026 | | 0.160±0.124 |
| Group | Porphyromonas  Mean SD | | Prevotella  Mean SD | | Rothia  Mean SD | | Streptococcus  Mean SD | | Veillonella  Mean SD | |
| H-H-T0 | 0.047±0.056 | | 0.187±0.128 | | 0.044±0.057 | | 0.147±0.089 | | 0.187±0.156 | |
| H-C-T0 | 0.022±0.024 | | 0.221±0.185 | | 0.106±0.089 | | 0.131±0.099 | | 0.122±0.064 | |
| H-H-T1 | 0.038±0.029 | | 0.101±0.082 | | 0.101±0.059 | | 0.093±0.065 | | 0.090±0.072 | |
| H-C-T1 | 0.012±0.008 | | 0.285±0.134 | | 0.079±0.087 | | 0.043±0.020 | | 0.187±0.067 | |
| H-H-T2 | 0.037±0.029 | | 0.231±0.156 | | 0.050±0.032 | | 0.093±0.048 | | 0.118±0.099 | |
| H-C-T2 | 0.012±0.013 | | 0.246±0.142 | | 0.047±0.039 | | 0.069±0.081 | | 0.222±0.137 | |
| H-H-T3 | 0.039±0.032 | | 0.205±0.161 | | 0.073±0.056 | | 0.097±0.072 | | 0.069±0.056 | |
| H-C-T3 | 0.026±0.024 | | 0.260±0.162 | | 0.068±0.041 | | 0.057±0.033 | | 0.191±0.108 | |
| H-H-T4 | 0.046±0.036 | | 0.187±0.162 | | 0.095±0.071 | | 0.165±0.077 | | 0.062±0.038 | |
| H-C-T4 | 0.031±0.039 | | 0.274±0.122 | | 0.076±0.043 | | 0.115±0.059 | | 0.137±0.082 | |
| H-H-ALL | 0.041±0.037 | | 0.184±0.143 | | 0.071±0.059 | | 0.121±0.076 | | 0.107±0.103 | |
| H-C-ALL | 0.022±0.026 | | 0.256±0.148 | | 0.078±0.065 | | 0.089±0.073 | | 0.164±0.095 | |
